# Patient perception of doctor communication skills and patient trust in rural primary health care: the mediating role of health service quality

**DOI:** 10.1186/s12875-022-01826-4

**Published:** 2022-09-29

**Authors:** Linni Gu, Bo Tian, Yujia Xin, Shengfa Zhang, Jing Li, Zhijun Sun

**Affiliations:** 1 School of Health Management, Inner Mongolia Medical University, Jinshan Development Zone, Hohhot City, Inner Mongolia 010110 China; 2 School of Social Development and Public Policy, Beijing Normal University, 19 Xinjiekou Wai Street, Haidian District, Beijing, 100875 China; 3 Business School, Beijing Normal University, 19 Xinjiekou Wai Street, Haidian District, Beijing, 100875 China

**Keywords:** Communication skills, Doctor-patient relationship, Trust, Health service quality, Rural health

## Abstract

**Background:**

This study aimed to explore the relationship between communication skills, health service quality, and patient trust in primary health services.

**Method:**

This study was conducted in village clinics in rural China. A simple random sampling method was used to select volunteer village clinics and patients. In total, 574 participants from 25 village clinics were selected with the help of local health officers and village doctors. The response rate was 90%. Statistical analyses (hierarchical linear regression analysis and a structural equation model) were performed to analyze the data.

**Results:**

Patient trust in doctors in rural primary health was influenced by patient perceptions of doctors’ communication skills and health service quality. However, health service quality fully mediated the relationship between doctors’ communication skills and patient trust in village clinics. In other words, doctors’ communication skills indirectly influence patients’ trust in doctors.

**Conclusions:**

This study found a link between doctors’ communication skills and patient trust. The findings suggest that health managers and doctors should attach great value to communication skills and health service quality in promoting the rural doctor-patient relationship. Moreover, the relationship between doctors and patients should be considered when reforming the primary health system.

**Supplementary Information:**

The online version contains supplementary material available at 10.1186/s12875-022-01826-4.

## Background

Given its association with treatment outcomes and patient satisfaction, the doctor-patient relationship is central to healthcare delivery [1] and can greatly improve patient health and well-being [2]. Moreover, a harmonious doctor-patient relationship is a safeguard for the health system. In China, however, the relationship between doctors and patients has become increasingly strained, with the country experiencing a surge in medical disputes in recent years. According to media reports, there were more than 12 violent injuries to doctors in 2018 [3]. Violence against doctors has become common and has drawn widespread concern from policymakers, medical professionals, and the general public [1].

Building a positive doctor-patient relationship has received significant attention worldwide. Numerous studies have explored the doctor-patient relationship from different perspectives. A previous study found that strained doctor-patient relationships have greatly influenced healthcare systems’ development and doctors’ passion and enthusiasm for work, using normative analysis [4]. Other studies have attributed the deterioration of doctor-patient relationships to the degeneration of doctor-patient trust using both normative and empirical analysis [5–8]. However, a great deal of evidence has confirmed that doctor-patient trust plays a pivotal role in this relationship [9–12] in China and other developed countries. “Patient trust doctor” means a patient trusts his doctor and believes that his doctor will consider his benefit and provide help and assistance concerning treatment and healthcare [13]. Several studies have indicated that a trusted doctor spares no effort to enhance patient adherence to treatment recommendations and clinical outcomes [14–16]. However, another study identified doctors’ communication skills as the core factor influencing the doctor-patient relationship [17]. Communication skills are related to the ability to transfer information effectively and efficiently. This is consistent with a set of skills, including verbal, nonverbal, attentive listening, emotional handling, and building trust [18]. Deficient communication can easily lead to medical disputes, negative medical experiences, malpractice litigation, and distrust in doctors [18]. Yudha et al. revealed that doctors’ communication skills influence cesarean section patients’ comprehension [19]. Roger et al. found that doctors’ communication skills influenced patient satisfaction and pointed out that doctors communicated less during treatment [20]. Harber et al. identified that doctor-patient communication impacts patients’ perception of health service quality [21]. Nevertheless, previous studies have also confirmed that good health service quality is associated with retaining current customers and attracting new ones, decreased costs, enhanced hospital image, and eventually, increased profitability [22, 23]. Similarly, other studies have shown that the quality of health services is strongly related to patient satisfaction and that patients consider the quality of health care to be good if they are satisfied [24, 25]. A follow-up study found that health service quality influences patient trust [26]. Overall, numerous studies have explored the relationship between communication skills and patient trust or the relationship between health service quality and patient trust. However, the association between doctors’ communication skills, health service quality, and patient trust in the doctor-patient relationship in primary healthcare is unknown, particularly in rural China.

In rural China, village clinics serve as the primary healthcare organization in the three-tiered rural public health system, where village doctors act as patients’ first line of contact with the health system [27]. Health workers in rural areas were mainly composed of “barefoot doctors” (health workers were recruited from the farmers, given limited training, and then provided health services before the 1980s), village doctors, and a small number of general practitioners (GP). However, rural primary health has faced many challenges, such as a lack of qualified general practitioners, poor service quality, and medical facilities [28]. Meanwhile, patients were reluctant to visit doctors in rural primary health. In 2013, the National Health Commission of the People’s Republic of China published the *Guidance on the Pilot Contract Services for Rural Doctors,* which required village doctors to change service models to promote public health and basic medicine in rural areas and attract patients.

Based on the above literature review and rural health condition analysis, the main objective of this study was to explore the pathway between patient perceptions of doctors’ communication skills, health service quality, and patient trust in doctors. Theoretically, doctors’ communication skills could affect patients’ perceptions of health service quality, influencing their trust in doctors. Specifically, this study explores whether doctors’ communication skills directly affect patients’ trust in doctors, with service quality acting as a mediator, and whether a direct or indirect association exists between communication skills and patients’ trust in doctors in the village clinics of rural China after controlling for demographics, medical expenditure, and other health policy characteristics. Therefore, we hypothesized that doctors’ communication skills positively influence patients’ trust in doctors in village clinics in rural China and that service quality mediates the pathway between doctors’ communication skills and patients’ trust in doctors.

## Methods

### Study design and setting

This cross-sectional quantitative study was conducted in the DF District, Jiangsu Province, WF County, Hubei Province, and YN County, Shandong Province. Regarding the geographic location, Jiangsu Province is located in China’s eastern part; Hubei Province lies in the central section of China; Shandong Province is situated in the north of Jiangsu Province. Regarding the economy, DF District was the richest of the three and had a GDP of 647.48 billion yuan in 2017 [29], with an annual per capita disposable income of rural residents of 21,202 yuan [30]. YN had a GDP of 262 billion yuan in 2017, with an annual per capita disposable income of rural residents of 12,105 yuan [31], and WF County had a GDP of 654,9 million yuan in 2017, with an annual per capita disposable income of rural residents of 9498 yuan. Twenty-five village clinics were selected with the help of local government officers at the town’s central hospital.

### Sampling

A multistage random sampling method was used for the sample selection. First, three provinces were chosen according to their geographic location and economy. Meanwhile, investigation feasibility and limited financial support were considered. Three counties were selected from each province. The town was randomly selected from the list provided by the local officer. Clinics were selected using the same method. We chose 8–9 village clinics in each county. Ultimately, twenty-five village clinics were selected. The participants were selected according to each village clinic’s daily outpatient visits and contract service lists. The sample size was calculated using the formula to ensure demographic representation (see formula (1)) [32], and approximately 504 participants had a sufficient sample size [11, 33]. Specifically, the actual population was assumed to be ±0.04 of the sample population. That is, the allowed error *e* = 0.04; the confidence level is 0.05 (two sides), *t* = 1.96, *p* = 0.5 (according to a previous study and textbook recommendation) [32, 34]. Meanwhile, the reality was also considered: the number of rural residents decreased due to industrialization and consequently the number of rural patients also declined. Patients who were working in the field during the investigation were excluded. The criteria for participant eligibility were as follows: had attended the clinic within the last 6 months, adult, had good hearing and mental health, and had no difficulty in speaking. Finally, 625 patients participated in the investigation by completing the questionnaire after providing informed consent. Each participant received a towel for participating in the investigation. Ultimately, 574 valid questionnaires were received, with a response rate of 90%.

1 $$n=\frac{t^2\times p\left(1-p\right)}{e^2}$$

### Data collection

Data collection was conducted from October to November 2017 in 25 village clinics in three counties with the help of the investigators. The selected participants were convened in the clinic meeting room to complete a structured questionnaire. The questionnaire was self-designed according to our study goal, and its validity was presented in previously published articles [11]. It mainly asked about the patients’ basic demographics, their trust in doctors, doctors’ communication skills, the clinic’s health service quality, the communication instrument, and satisfaction with drug utilization. These variables were measured separately using the Wake Forest Physician Trust Scale (WFPTS), the TCom-skills GP Scale (communication skills), the ServQual (service quality) scale, and category variables. Four investigators from Beijing Normal University helped complete the investigation. All investigators were rigorously trained for 2 weeks by experts and appropriately qualified for questionnaire delivery, and quality supervisors reviewed all completed questionnaires after each interview.

### Data collection statement

All the methods above were performed in accordance with the Declaration of Helsinki.

### Measurement and variables

#### Demographic information

Eligible participants were assessed in village clinics by using a structured questionnaire. Demographic and socioeconomic information were collected, including age, sex, educational level, and economic status. Specifically, economic status was divided into three levels according to local annual per capita wage income (see Table 1).

**Table 1:** Sample characteristics

Variables	*N* (574)	Percentage (%)
Gender		
Male	315	54.88
Female	259	45.12
Age		
≤ 40	72	12.54
41–59	264	45.99
≥ 60	238	41.46
Education		
Primary school or below	184	32.06
Junior high school	254	44.25
Senior high school or above	136	23.69
Family income (Yuan)		
≤ 9999	173	30.14
10,000–29,999	290	50.52
≥ 30,000	111	19.34
Satisfaction with drug supply of village clinic		
Yes	384	66.90
No	190	33.10
Satisfaction with drug treatment effect		
Bad	19	3.31
Good	555	96.69
Communication instrument		
QQ/Weichat		
Yes	187	32.58
No	387	67.42
Short message		
Yes	226	39.37
No	348	60.63
Clinic (*numbers*)	25	

#### Other control variables

Other variables that might influence patients’ trust in doctors were included in this study. Drug utilization satisfaction was measured using the following questions:(1) Do village clinics provide the drugs you need? Answers were dichotomized as No = “0″ and Yes = “1″; (2) What do you think of the drug effects of the village clinics? Answers were “bad, good.” The communication instrument was measured using the following questions: (1) Do you use WeChat and QQ to communicate with village doctors? (2) Do you use short messages to communicate with the village doctors? Answers were dichotomized as “yes = 1″ and “no = 0.”

#### Communication skills

The translated version of the TCom-skills GP Scale was used to measure village doctors’ communication skills as assessed by the patients. This scale is widely used in the West to measure general practitioner communication skills in primary health services. The village doctor takes responsibility for general practitioners in primary healthcare in these rural areas. It consists of 15 items rated on a semantic differential scale ranging from 0 (never) to 7 (always) (see Table 2). Translation and back-translation were used to create a Chinese version of the scale. Internal coherence reliability was high, with a Cronbach’s α of 0.92 [35].

**Table 2:** Description of TCom-skills GP scale

Items	Obs	Mean	*SD*	Min	Max
1.Does the general practitioner take time to listen to me?	574	5.90	1.36	1	7
2. Does everything make me feel I can trust him/her?	574	5.50	1.32	1	7
3. Does the general practitioner explain what the treatment is for?	574	5.62	1.38	1	7
4. Does the general practitioner take account of my preferences in prescribing medication?	574	5.68	1.41	1	7
5. Does the general practitioner give me the impression he/she has respect for me?	574	5.89	1.54	1	7
6. Does the general practitioner give me information on the side effects of medication?	574	5.65	1.48	1	7
7. Does the general practitioner emphasize which are the most important drugs?	574	6.00	1.11	1	7
8. Does the general practitioner discuss any difficulties I have in complying with the treatment?	574	5.06	1.82	1	7
9. Does the general practitioner explain things in simple words? 10. Does the general practitioner offer new treatments?	574 574	5.71 5.58	1.43 1.49	1 1	7 7
11. Does the general practitioner write the prescription legibly?	574	5.43	1.62	1	7
12. Does the general practitioner let me ask questions?	574	5.91	1.33	1	7
13. Does the general practitioner give me incentives to comply with the treatment?	574	5.90	1.29	1	7
14. Does the general practitioner give me advice on prevention (diet, physical activity)?	574	6.05	1.18	1	7
15. Does the general practitioner give the impression he/she knows his/her job?	574	5.93	1.31	1	7
Total score		5.72	1.40		

#### Service quality

A modified SERVQUAL Scale was used to measure health service quality at village clinics. It includes 22 items that measure the five dimensions of health service quality. Each item is scored from 1 (very bad) to 9 (very good). The scale’s reliability was shown by a Cronbach’s α of 0.955 in reliability analysis.

#### Trust

The Chinese version of the Wake Forest Physician Trust Scale (WFPTS), a commonly used scale in China, was adopted to evaluate patient trust in doctors [11, 36]. It includes 10 items; each scored on a 5-point Likert scale. The reliability of the scale (Cronbach’s α) was 0.76.

#### Clinic

A previous study suggested that environmental factors affect individual behavior [37]. Therefore, this study considered the clinic an environmental variable and added it to the model.

### Statistical analysis

The following analytic steps were employed to explore the relationship between patients’ perception of doctors’ communication skills and patients’ trust in doctors. First, descriptive analysis and normal distribution tests were adopted to understand the sample’s overall characteristics and diagnose the model. Second, Pearson’s correlation coefficients were calculated to examine pairwise associations between communication skills, health service quality, and trust. Hierarchical linear regression analysis was then employed to verify the relationships between communication skills, health service quality, and trust after controlling for confounders (gender, identity, age, marital status, education, and family income) [34, 38]. The clinic was controlled as an environmental variable and added to the model. The Sobel-Goodman mediation test was adopted in the following step to examine the proposed model [39–41]. Third, pathway CS → SQ → T (see Model 2) analysis was performed. Specifically, the independent variable communication skills (CS) and the dependent variable trust (T) were entered in the first regression model. In the second model, service quality was the dependent variable, and the model examined whether communication skills influenced the quality of service; independent variables (CS, SQ) and the dependent variable (T) were fully entered in the third model. In the model, a, b, and c are the regression coefficients of interest for comparison; d, f, and g are intercepts; and e1, e2, and e3 are error terms. SQ is considered to completely mediate the relationship between CS and T if a, c, and b are statistically significant but c’ is not significant. SQ was considered a partial mediating effect if c’ was statistically significant but smaller than c. The above analyses were implemented using the Stata software package (version SE15, Stata Corp., College Station, TX, USA).

2 $${\displaystyle \begin{array}{c}T=d+ cCS+e1\\ {} SQ=f+ aCS+e2\\ {}T=g+{c}^{'} CS+ bSQ+e3\end{array}}$$

The regression model provides a straightforward relationship between the third variables but does not offer a goodness-of-fit between the data and hypothesized pathway. To further confirm our hypothesis, structural equation modeling was performed using Mplus 8.3. (developed by Muthen and Muthen). The goodness-of-fit indices were χ^2^/df = 0.10, RMSEA = 0.01, SRMR = 0.01, CFI = 0.99, and TLI = 0.99. Two-tailed *P* values < 0.05 were considered statistically significant.

## Results

### Participant characteristics

A total of 574 patients participated in the study, with slightly more males (315, 54.88%) than females (259, 45.12%). Regarding age, a large number of participants were between 40 and 59 years, more than 41% were over 60 years, and a small portion was under 40 years. Regarding education, most students did not study beyond high school. More than half of the participants had an annual family income of less than 30,000RMB (see Table 1).

### Patient perception of doctors’ communication skills

Table 2 summarizes patients’ perceptions of doctors’ therapeutic communication skills in village clinics. The results demonstrated that the mean scores for each item were high. The total average communication score was 5.72 (SD = 1.40; range:1–7).

### Service quality

Table 3 presents the results of patient perceptions of village clinics’ service quality. The results demonstrated that the service quality scores of the separated items were high, with an average score of 7.08 (SD = 1.67; range:1–9).

**Table 3:** Description of ServQual

Items	Obs	Mean	SD	Min	Max
1. The environment of the clinic is comfortable, clean and convenient. 2. The clinic is equipped with matching medical equipment. 3. The clinic’s service window is reasonable layout. 4. Village doctor dress neatly and professionally. 5. The treatment process of the clinic is simple and convenient. 6. Village doctor care and help you when you are in illness. 7. The village doctor measures your blood pressure, blood glucose, and gives health promotion regularly.	574 574 574 574 574 574 574 574	7.14 5.86 6.71 7.18 7.12 7.12 7.33 7.20	1.88 2.38 2.08 1.85 2.06 2.06 1.98 1.98	1 1 1 1 1 1 1 1	9 9 9 9 9 9 9 9
8. You can contact the village doctor easily and quickly.	574	7.31	1.93	1	9
9. The village doctor can record your information correctly and maintain your privacy.	574	7.53	1.74	1	9
10. The village doctor diagnoses accurately and controls treatment costs.	574	6.83	2.28	1	9
11. The village doctor provides a timely service and tells you the exact service time.	574	6.98	2.01	1	9
12. The village doctor pays more attention to the patient’s suggestions, opinions, and complaints.	574	6.77	2.11	1	9
13. The village doctor helps you immediately and satisfactorily even if he (she) is busy.	574	7.01	1.93	1	9
14. The village doctor can contact other doctors to assist when he/she cannot provide a specific service.	574	7.21	2.10	1	9
15. The village doctor get you and your family’s permission before performing special examination or treatment.	574	7.53	1.57	1	9
16. The village doctor uses medical instruments professionally, and makes you feel at ease.	574	7.24	1.75	1	9
17. The village doctor has a strong service delivery attitude.	574	7.42	1.74	1	9
18. The village doctor is happy to explain the illness and directs you to take medicine carefully and patiently.	574	7.38	1.71	1	9
19.The village doctor provides personalized care for contracted families.	574	6.78	2.16	1	9
20.The village doctor knows your family and health status.	574	6.97	2.19	1	9
21. The village doctor considers your interest first and eases your trouble.	574	7.17	1.87	1	9
22. The service hours of the village clinic meet your requirements.	574	7.09	1.99	1	9
Total score		7.08	1.67		

### Trust

Table 4 summarizes the results of patient trust in doctors and the normal tests of the dependent variables. The results demonstrated that each item’s mean score was high, with an average score of 3.53 (SD = 0.51; range:1–5). The normal test value for skewness was 0.08, and kurtosis was 2.92.

**Table 4:** Description of trust

Items	Obs	Mean	SD	Mix	Max
1. Your doctor cares about your health just as much or more than you do.	574	3.95	0.73	1	5
2. Sometimes doctors care more about what is convenient for them than about their patients’ medical needs.	574	2.78	1.26	1	5
3. Doctors’ medical skill are not as good as they should be.	574	3.11	0.76	1	5
4. Your doctor is extremely thorough and careful.	574	4.15	0.77	1	5
5. You completely trust your doctor’s decisions about which medical treatments are best for you.	574	3.91	0.77	1	5
6. Your doctor is totally honest in telling you about all the different treatment options available for your condition.	574	3.80	0.88	1	5
7. Sometimes doctors do not pay full attention to what patients are trying to tell them.	574	2.49	1.16	1	5
8. Your doctor only thinks about what is best for you.	574	3.83	1.00	1	5
9. You have no worries about putting your life in your doctor’s hands.	574	3.30	1.38	1	5
10. All in all, you trust doctors completely.	574	4.02	0.78	1	5
Total score		3.53	0.51		
Skewness	0.08				
Kurtosis	2.92				
*P* (normal)	0.95 > 0.05				

### Correlations between communication skills, service quality, and trust

The Pearson’s correlation coefficient was used to test the associations between the three study variables. As shown in Table 5, communication skills, service quality, and trust are highly correlated. Communication skills and service quality were positively associated with trust.

**Table 5:** Pairwise correlations between communication, health service quality, and trust

	communication	service quality	trust
Communication	1		
Service quality	0.480^***^	1	
Trust	0.582^***^	0.702^***^	1

^∗∗∗^*P* < 0.01

### The mediating effects of service quality on the association between communication skills and trust

**Fig. 1:**
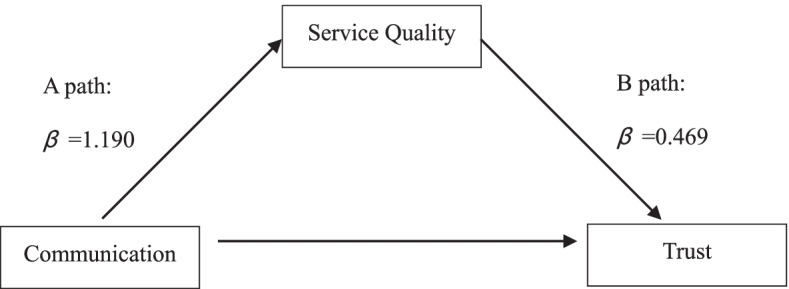
Service quality as a mediator between communication and trust. Note: A-path: the independent variable (communication) significantly influences the dependent variable (trust) in the absence of the mediator (service quality). B-path: the mediator (service quality) has a significant unique effect on the dependent variable (trust), and means a direct effect of service quality on the dependent variable (trust). C-path: the independent variable (communication) significantly influence the dependent variable (trust). C′-path: the independent variable (communication) does not influence the dependent variable directly (trust) when adding the mediator (service quality). Model goodness-of-fit statistics: χ^2^/df = 0.10, RMSEA = 0.01, SRMR = 0.01, CFI = 0.99, TLI = 0.99

The results of the Sobel-Goodman mediation tests (*P* = 0.000) indicated that an indirect link exists between doctors’ communication skills and patient trust after controlling for confounders. Table 6 and Fig. 1 summarize the regression results from doctors’ communication skills to patients’ trust based on service quality. The results demonstrate that service quality fully mediates the relationship between communication skills and trust. As shown in Fig. 1, (1) Path A shows the direct effect of communication skills on service quality (*β* = 1.190, *P* < 0.001, CI:1.11,1.27); (2) Path B represents the direct effect of service quality on trust (*β* = 0.469, *P* < 0.001, CI:0.41,0.53); (3) Path C represents the direct effect of communication skills on trust (*β* = 0.378, *P* < 0.001, CI:0.31,0.44); and (4) Path C represents the pathway of communication skills and service quality on trust (*β* = 0.437, *P* < 0.001, CI: − 0.06,0.14). The results confirm the hypothesized mediating role of service quality between communication skills and patient trust in doctors.

**Table 6:** Mediation effect between Communication and Trust

	A path(β)	B path(β)	C path(β)	C′ path(β)
	Direct effects of communication on service quality	Direct effects of service quality on trust	Direct effects of communication on trust	Coeffect of communication and service quality
Communication	1.190^***^		0.378^**^	0.041
Service quality		0.469^***^		0.437^***^
Cons	−0.241^***^	−0.335^***^	−0.485^**^	−0.337^***^
*P* 95% CI	< 0.001 (1.11, 1.27)	< 0.001 (0.41, 0.53)	< 0.001 (0.31, 0.44)	< 0.001 (−0.06, 0.14)
*Sobel test*				*P* = 0.000

Note: (1) standard errors in parentheses; ^***^P < 0.01; ^**^P < 0.05; ^*^P < 0.1

(2) Mediation effect calculation: 𝐸 = (A path) ∗ 𝛽(B path)/𝛽(C path) = (1.190) ∗ (0.469)/0.378 = 147.6%

(3) Mediating effect of total effect calculation: 𝑃 = (Mediation Effect)/(𝐸(Mediation Effect) + 𝛽(C path)) = 147.6%/(147.6% + 37.8%) = 79.6%

## Discussion

To the best of our knowledge, this is the first study to explore the mediating pathway between communication skills and trust in China’s primary health care system. This study aimed to clarify the relationship between these three variables in the health service system. This finding will help doctors and patients understand the importance of trust in establishing a doctor-patient relationship. It will also help doctors attach great importance to communication skills and health services for their regular diagnosis and treatment.

In this study, we found that communication was significantly associated with trust in the absence of service quality. This is consistent with a previous study on the relationship between patient-doctor communication and trust among older adult Medicare beneficiaries in the United States [42]. Effective communication is essential for increasing patient trust in doctors. Quirk et al. pointed out that doctors who had a hurried attitude during their visits were perceived by patients as uncaring [43]. Patients preferred doctors who spent sufficient time with them to discuss their illness and symptoms. This was particularly the case for older adult patients who needed doctors to carefully and clearly explain their symptoms and drug use. Rainer et al. confirmed that empathy, reassurance and support, encounter length, history taking, and psychosocial talk as verbal communication played significant roles in disease treatment [44]. This study also found that emphasizing the most important drugs, explaining the treatment, respecting patients, and providing incentives to comply with the treatment can efficiently decrease patients’ tension and enhance their trust in doctors.

The most important contribution of this study was the finding that service quality fully mediated the association between patients’ perception of doctors’ communication skills and patients’ trust in doctors. In this study, we found that patients’ perception of doctors’ communication skills did not directly influence their trust in doctors when considering health service quality. Patients’ perceptions of doctors’ communication skills influenced their trust via doctors’ quality of service. According to previous studies, service quality plays a determining role in establishing trust and creating a relatively strong relationship between patients and doctors. Ehsan et al. confirmed that good health service providers do not work for their own benefit and do not take actions detrimental to patients [26]. Another author, Dini Ghaliyah, indicated that the core element of service quality was to satisfy patients’ needs and expectations, which would result in patients having a positive perception of doctors’ capability [45]. In other words, patients’ needs and expectations were satisfied through doctor-patient communication.

Regarding the relationship between rural doctors and patients, rural patients in China see village doctors mainly because they believe in their quality of service. In particular, they considered that doctors’ empathy was the most important factor in health services (mean value:35.41). However, village doctors provide basic health care services and take care of issues such as colds, diarrhea, and stomachaches. They also provide villagers with public health care, health prevention, and rehabilitation. If a patient’s illness exceeds their service scope and capability, they must help patients by referring them to higher-level hospitals. That is, village doctors provide not only basic medicine to villagers but also non-medical services. In this service model, doctors’ communication skills play a vital role in basic medical and nonmedical services. Irmak et al. asserted in their study that doctor-patient communication is the foundation of healthcare services [46]. Patient perceptions of doctors’ quality of healthcare emanate from their perceptions of doctors’ communication skills. An earlier study revealed that effective communication increases patients’ perceptions of doctors’ health service quality [47]. Similarly, our study found that patients who scored higher on the perception of doctors’ communication skills (mean value:5.90) also scored higher on patients’ perception of doctors’ healthcare service quality (mean value:7.08). The reason was that doctors explained the illness and treatment clearly and carefully, thus eliminating patients’ uncertainties, fears, and anxieties. Similarly, using plain words instead of professional terms would facilitate patients’ understanding of their treatment and improve their trust in their doctors. Doctors who patiently and carefully listen to patients, discuss their illness, and obtain more information about the illness than doctors who do not appear to be more caring. The descriptive analysis showed that many patients were older adults and had lower educational attainment, indicating that village doctors need more patience and use simpler words rather than professional terms to communicate with patients. This explains why rural patients’ perceptions of doctors’ communication skills are good, and they consider their service quality to be high, further influencing the doctor-patient trust relationship. A previous empirical study indicated that health service quality is strongly linked to patient trust in doctors [22]. Another study confirmed that patient trust in doctors increased when doctors satisfied their needs and expectations [26]. Our study also found that health service quality positively influences patients’ trust in doctors. Overall, village doctors’ communication skills increase patients’ perceptions of health service quality and enhance patients’ trust in doctors. Our findings reconfirmed that doctors’ communication skills are the most important elements in establishing or promoting doctor-patient relationships.

This study’s findings on patient trust in doctors will contribute to a growing body of evidence for improving doctors’ communication skills and health service quality. It will also draw much attention to patients’ trust in doctors during the COVID-19 pandemic. As highlighted above, conflicts exist between doctors and patients in hospitals worldwide. Although numerous studies have confirmed that improving doctors’ communication skills is conducive to improving doctor-patient relationships, this study’s findings link doctors’ communication skills and patient trust in analyzing doctor-patient relationships.

## Limitations

This study had several limitations. First, the sample size was small, and all participants came from one area; therefore, the results cannot be extrapolated to other counties, provinces, or the whole of China. Second, causality inferences were not made between the variables because of the cross-sectional nature of the data. Third, communication and trust were interaction behaviors in the disease treatment process; this study explored this relationship from the patients’ perspective. Thus, interactional relationships between them should be examined in the future. Finally, this study explored the mediating role of health service quality, and whether communication skills played a mediating role was unknown, which will be explored in a future study.

## Conclusions

To our knowledge, this is the first study to explore the indirect influence of communication skills on patient trust through health services quality. The results indicate that health service quality fully mediated this relationship. Communication skills influence patient trust through service quality, which shows that service quality is a critical element of the health service system. Therefore, doctors or health providers in primary healthcare systems should improve the quality of health services. Meanwhile, this study’s findings contribute to a broad body of literature exploring the function of health service quality in doctor-patient relationships.

The study’s findings strengthen the importance of primary health in rural China reform and provide a way to implement comprehensive health policy, including sustainable health training programs. With the increase in the number of aging people in China and the implementation of the rural revitalization policy, rural doctors’ communication skills will play a crucial role in enhancing doctor-patient relationships, improving patients’ experience in health services, and influencing rural residents’ well-being.

## Supplementary Information

**Additional file 1.**

## Data Availability

The dataset is available from the corresponding author, who will provide it on a reasonable request.

## Abbreviations

GP: General Practitioner
TCom-skills: Therapeutic Communication Skills
WFPTS: Wake Forest Physician Trust Scale
ServQual: Service Quality
