# Supplementary material for: Patient perception of doctor communication skills and patient trust in rural primary health care: the mediating role of health service quality
Source: BMC Prim Care. 2022 Sep 29;23:255. doi: 10.1186/s12875-022-01826-4 (PMC9520094; doi:10.1186/s12875-022-01826-4)
Supplement: Supplementary file 1 — Additional file 1. [file 12875_2022_1826_MOESM1_ESM.docx]

**Rural Doctor-Patient Relationship Questionnaire**

1. **Demographic characteristics**
2. Your gender is?

①male ②female

1. Your occupation is?

①farmer ②worker

1. Your age is?

①≤40 ②41-49 ③50-59 ④60－74 ⑤≥75

1. Your martial status is?

①married ② single

1. Your education background is?

①illiteracy ②primary school ③ junior school ④high school ⑤college

1. In the past year, your annual household income is?

①≤ 9,999 ②10,000–29,999 ③≥ 30,000

1. **According to your understanding, please answer questions as follows:**
2. Do village clinics provide drugs you need?? ①Yes ②No
3. What do you think of the drug effects of the village clinics? ①Bad ②Good
4. Do you use QQ/Weichat to communicate with doctor? ①Yes ②No
5. Do you use short message to communicate with doctor? ①Yes ②No
6. **Please evaluate doctor’s communication skill according to your recent clinic experience.**

| 1. Does the general practitioner take time to listen to me?  never always  1 2 3 4 5 6 7 |
| --- |
| 2. Does everything make me feel I can trust him/her?  never always  1 2 3 4 5 6 7 |
| 3. Does the general practitioner explain what the treatment is for?  never always  1 2 3 4 5 6 7 |
| 4. Does the general practitioner take account of my preferences in prescribing medication?  never always  1 2 3 4 5 6 7 |
| 5. Does the general practitioner give me the impression he/she has respect for me?  never always  1 2 3 4 5 6 7 |
| 6. Does the general practitioner give me information on the side effects of medication?  never always  1 2 3 4 5 6 7 |
| 7. Does the general practitioner emphasize which are the most important drugs?  never always  1 2 3 4 5 6 7 |
| 8. Does the general practitioner discuss any difficulties I have in complying with the treatment?  never always  1 2 3 4 5 6 7 |
| 9. Does the general practitioner explain things in simple words?  never always  1 2 3 4 5 6 7  10. Does the general practitioner offer new treatments?  never always  1 2 3 4 5 6 7 |
| 11. Does the general practitioner write the prescription legibly?  never always  1 2 3 4 5 6 7  12. Does the general practitioner let me ask questions?  never always  1 2 3 4 5 6 7  13. Does the general practitioner give me incentives to comply with the treatment?  never always  1 2 3 4 5 6 7  14. Does the general practitioner give me advice on prevention (diet, physical activity)?  never always  1 2 3 4 5 6 7  15. Does the general practitioner give the impression he/she knows his/her job?  never always  1 2 3 4 5 6 7 |

1. **Please evaluate doctor service quality according to your recent clinic experience.**

| 1. The environment of the clinic is comfortable, clean and convenient.  very bad very good  1 2 3 4 5 6 7 8 9  2. The clinic is equipped with matching medical equipment.  very bad very good  1 2 3 4 5 6 7 8 9  3. The clinic’s service window is reasonable layout.  very bad very good  1 2 3 4 5 6 7 8 9  4. Village doctor dress neatly and professionally.  very bad very good  1 2 3 4 5 6 7 8 9  5. The treatment process of the clinic is simple and convenient.  very bad very good  1 2 3 4 5 6 7 8 9  6. Village doctor care and help you when you are in illness.  very bad very good  1 2 3 4 5 6 7 8 9  7. The village doctor measures your blood pressure, blood glucose, and gives health promotion regularly.  very bad very good  1 2 3 4 5 6 7 8 9 |
| --- |
| 8. You can contact the village doctor easily and quickly.  very bad very good  1 2 3 4 5 6 7 8 9 |
| 9. The village doctor can record your information correctly and maintain your privacy.  very bad very good  1 2 3 4 5 6 7 8 9 |
| 10. The village doctor diagnoses accurately and controls treatment costs.  very bad very good  1 2 3 4 5 6 7 8 9 |
| 11. The village doctor provides a timely service and tells you the exact service time.  very bad very good  1 2 3 4 5 6 7 8 9 |
| 12. The village doctor pays more attention to the patient’s suggestions, opinions, and complaints.  very bad very good  1 2 3 4 5 6 7 8 9 |
| 13. The village doctor helps you immediately and satisfactorily even if he (she) is busy.  very bad very good  1 2 3 4 5 6 7 8 9 |
| 14. The village doctor can contact other doctors to assist when he/she cannot provide a specific service.  very bad very good  1 2 3 4 5 6 7 8 9 |
| 15. The village doctor get you and your family's permission before performing special examination or treatment.  very bad very good  1 2 3 4 5 6 7 8 9 |
| 16. The village doctor uses medical instruments professionally, and makes you feel at ease.  very bad very good  1 2 3 4 5 6 7 8 9 |
| 17. The village doctor has a strong service delivery attitude.  very bad very good  1 2 3 4 5 6 7 8 9 |
| 18. The village doctor is happy to explain the illness and directs you to take medicine carefully and patiently.  very bad very good  1 2 3 4 5 6 7 8 9 |
| 19.The village doctor provides personalized care for contracted families.  very bad very good  1 2 3 4 5 6 7 8 9 |
| 20.The village doctor knows your family and health status.  very bad very good  1 2 3 4 5 6 7 8 9 |
| 21. The village doctor considers your interest first and eases your trouble.  very bad very good  1 2 3 4 5 6 7 8 9 |
| 22. The service hours of the village clinic meet your requirements.  very bad very good  1 2 3 4 5 6 7 8 9 |
|  |

1. **According to your recent clinic experience, please answer question as follows:**

| Items | strongly disagree | disagree | uncertainty | agree | strongly agree |
| --- | --- | --- | --- | --- | --- |
| 1. Your doctor cares about your health just as much or more than you do. | 1 | 2 | 3 | 4 | 5 |
| 2. Sometimes doctors care more about what is convenient for them than about their patients’ medical needs. | 1 | 2 | 3 | 4 | 5 |
| 3. Doctors’ medical skill are not as good as they should be. | 1 | 2 | 3 | 4 | 5 |
| 4. Your doctor is extremely thorough and careful. | 1 | 2 | 3 | 4 | 5 |
| 5. You completely trust your doctor’s decisions about which medical treatments are best for you. | 1 | 2 | 3 | 4 | 5 |
| 6. Your doctor is totally honest in telling you about all the different treatment options available for your condition. | 1 | 2 | 3 | 4 | 5 |
| 7. Sometimes doctors do not pay full attention to what patients are trying to tell them. | 1 | 2 | 3 | 4 | 5 |
| 8. Your doctor only thinks about what is best for you. | 1 | 2 | 3 | 4 | 5 |
| 9. You have no worries about putting your life in your doctor’s hands. | 1 | 2 | 3 | 4 | 5 |
| 10. All in all, you trust doctors completely. | 1 | 2 | 3 | 4 | 5 |

**Thank you for your cooperation!**
